# An investigation into the use of < 38 µm fraction as a proxy for < 10 µm road dust particles

**DOI:** 10.1007/s10653-019-00350-2

**Published:** 2019-06-13

**Authors:** Andrew D. Brown, Judith E. S. Barrett, Michael Bennett, Sanja Potgieter-Vermaak

**Affiliations:** 1 School of Science and the Environment, Manchester Metropolitan University, Manchester, M1 5GD UK; 2 Molecular Science Institute, University of the Witwatersrand, Johannesburg, South Africa

**Keywords:** Size-segregated road dust, Respirable particles, Representation, Bioaccessibility

## Abstract

It is well documented that a large portion of urban particulate matters is derived from road dust. Isolating particles of RD which are small enough to be inhaled, however, is a difficult process. In this study, it is shown for the first time that the < 38 µm fraction of road dust particles can be used as a proxy for road dust particles < 10 µm in bioaccessibility studies. This study probed similarities between the < 10 and < 38µm fractions of urban road dust to show that the larger of the two can be used for analysis for which larger sample masses are required, as is the case with in vitro analysis. Road dust, initially segregated to size < 38 µm using sieves, was again size segregated to < 10 µm using water deposition. Both the original < 38 µm and the separated < 10 µm fractions were then subject to single particle analysis by SEM–EDX and bulk analysis by ICP-OES for its elemental composition. Dissolution tests in artificial lysosomal fluid, representative of lung fluid, were carried out on both samples to determine % bioaccessibility of selected potentially harmful elements and thus probe similarities/differences in in vitro behaviour between the two fractions. The separation technique achieved 94.3% of particles < 10 µm in terms of number of particles (the original sample contained 90.4% as determined by SEM–EDX). Acid-soluble metal concentration results indicated differences between the samples. However, when manipulated to negate the input of Si, SEM–EDX data showed general similarities in metal concentrations. Dissolution testing results indicated similar behaviour between the two samples in a simulated biological fluid.

## Introduction

Urban air quality is of significant importance to the majority of us living or working in our cities worldwide. Short-term events such as the 1952 London smog episode have the ability to dominate headlines because of the 4000 deaths due to acute exposure to air pollution, 12,000 when integrated over the whole of the winter (Whittaker et al. 2004). Chronic exposure to air pollution, to which we are all subject, provides equally shocking statistics. The European Environment Agency estimates that poor air quality is responsible for 467,000 premature deaths per year amongst Europeans (EEA 2016). The World Health Organization estimates that air pollution is responsible for 8% of lung cancer deaths, 5% of cardiopulmonary deaths and 3% of respiratory infection deaths (WHO 2009).

Such deleterious effects on the population are often attributed to particulate matter (PM). Emission sources of PM are numerous and commonly include industrial and commercial output, vehicular emissions and wear, abrasion of road surfaces, etc. (Wang et al. 2016). Another large source of PM is believed to be resuspended road dust (RD), which can account for as much as 74% of total suspended particles by mass (Hien et al. 1999; Harrison et al. 1997) and has also been observed to be the largest and second largest contributor to PM_10_ and PM_2.5,_ respectively (Landis et al. 2017). Emphasis on RD as possibly the most significant contributor to PM will continue to grow because of difficulty regulating this substance and its many contributing materials, unlike vehicle exhaust emissions (Padoan et al. 2017).

Because of the range and nature of sources, PM should be considered unique for any given location. For that reason, PM is a topic which has been studied extensively, although recent consensus amongst the scientific community indicates that the micro-chemical structure of PM is responsible for the degree of toxicity (Rohr et al. 2011).

Due to the nature of PM, and particularly respirable PM_10_, it can be difficult to analyse micro-chemical structure, without specialised techniques such as micro-Raman spectroscopy (Worobiec et al. 2010) or surface-enhanced Raman spectroscopy (SERS) (Tian et al. 2014). There are further difficulties in obtaining a suitable sample mass with a view to carry out robust bulk in vitro studies, since common methods for modelling either the respiratory or the gastrointestinal route typically use upwards of 0.5 g per sample, including replicates (Colombo et al. 2008; Wragg et al. 2011). Collection on filters using high-volume particulate matter (PM10) samplers typically yields samples of less than 0.1 g (Shahsavani et al. 2012; Behrooz et al. 2017).

The aim of this study is to see if < 38 µm fraction of RD is analogous to the < 10 µm fraction, and thus assess the possibility of using the larger, more abundant size fraction of RD as a proxy for metals in airborne respirable particles originating from RD. This will be carried out using metal concentrations as a marker. A water deposition technique can be used to separate the RD fraction of < 10 µm from the original < 38 µm sample. The < 38 µm sample will also be soaked in deionised water to simulate the conditions to which the < 10 µm fraction was exposed, to allow for both samples to lose some water-soluble components in the process. Both fractions the < 10 µm and the < 38 µm will then be acid digested to compare their elemental profiles and exposed to artificial lung fluid to compare mobility of metals within the body. RD used in the study was collected in 2014 from Oxford Road, Manchester. The collection technique and handling of RD have previously been described by Brown et al. (2015).

## Methods

### <10 µm particle separation

Size separation was carried out using a sedimentation-in-water process based on the principles of Stokes’ law, which gives the velocity of a sphere in a viscous fluid under the force of gravity. Equation 1 is Stokes’ law, where *V* is velocity of a particle; *g* is acceleration due to gravity; *a* is the diameter of a particle (cm); *d*_1_ is the density of a particle; *d*_2_ is the density of water; *µ* is the viscosity of water (Haynes 2018). Mean density of particles was measured to be 3.2 g/cm^3^, as determined by a water displacement technique similar to Blake and Hartage (1986).

Stokes’ law

1 $$V = \frac{{2ga^{2} (d_{1} - d_{2} )}}{9\mu }$$

**Fig. 1:**
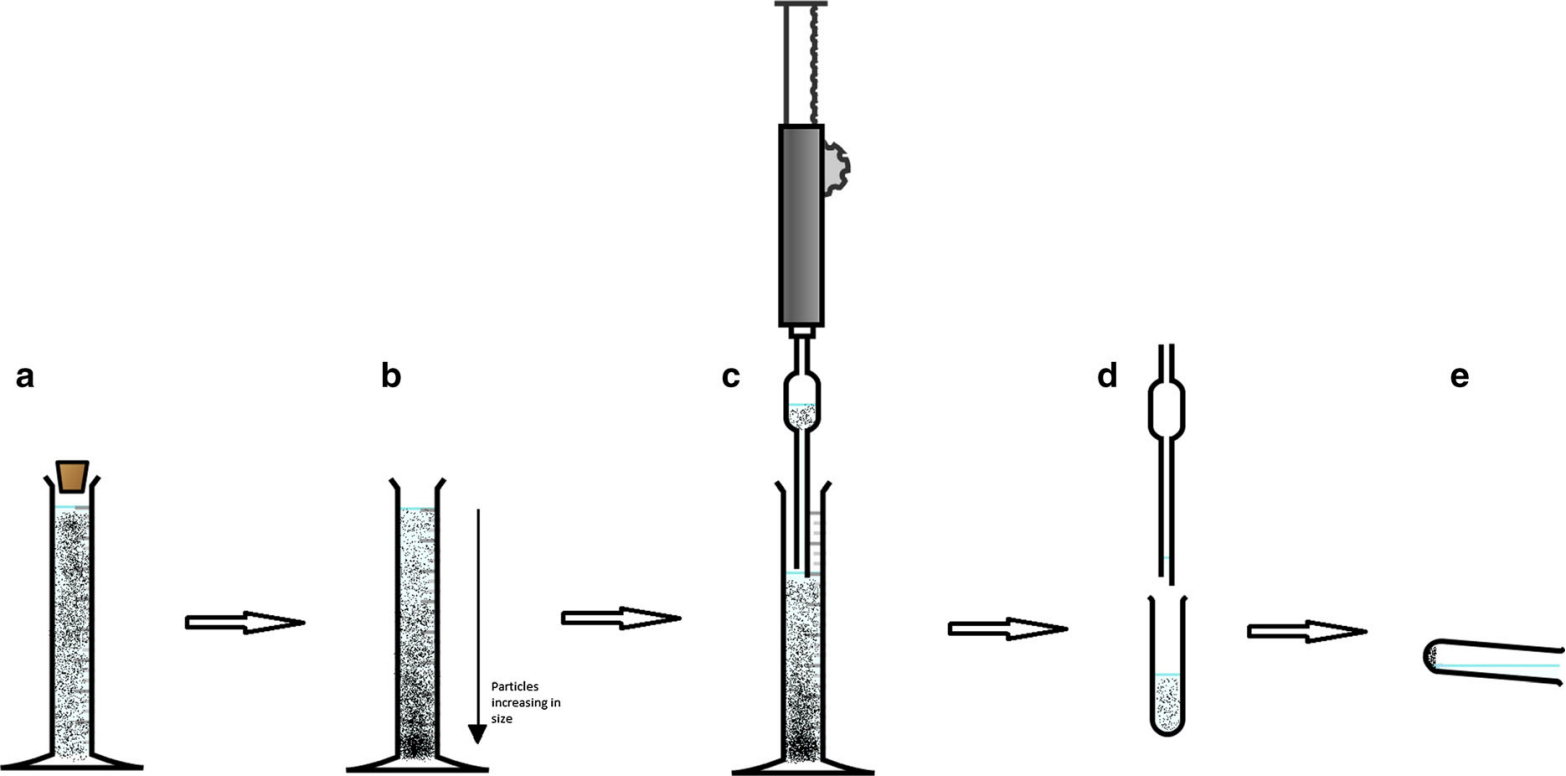
Separation schematic (https://chemix.org/)

The separation technique is presented in Fig. 1. The process uses a sealed 100 cm^3^ measuring cylinder. The distance between the 100 cm^3^ mark on the cylinder and the 80 cm^3^ mark was measured to be 3.1 cm. The time taken for mean density (*d*_1_) particles of 10 µm to travel that distance is 259 s, as determined by Eq. 1.

A 2 g sample of RD was accurately weighed into the measuring cylinder and then made up to the 100 cm^3^ mark with deionised water (18 MΩ). The cylinder and contents were thoroughly agitated by hand and then placed on a bench, and this is shown as step a. in Fig. 1. A stopwatch was then used to time 259 s, the amount of time taken for particles larger than 10 µm to travel 3.1 cm, shown as step b. in Fig. 1. A 20 cm^3^ pipette was then inserted into the cylinder to siphon off the top 20 cm^3^ of water. This process removes suspended particles < 10 µm from the cylinder, shown as step c. in Fig. 1. The water in the pipette, containing the < 10 µm particles, is then aspirated into a clean centrifuge vial, shown as step d. in Fig. 1. The centrifuge vial and contents were then centrifuged at 2000 rpm for 15 min, and the supernatant was then discarded, shown as step e. in Fig. 1. The particles remaining in the centrifuge tube were transferred to a clean weighing boat and left to dry at room temperature in a clean brown paper bag. This process is repeated 20 times for each sample with another 20 cm^3^ of deionised water added to the measuring cylinder before step a. to replace the 20 cm^3^ removed at step c. on the previous iteration. Twenty extractions were carried out as preliminary testing indicated this was the amount needed for the water in the pipette to appear to be visibly clear of suspended particles. The method described here is based on one described by Boisa et al. (2014).

### Particle size analysis

A Zeiss Supra 40VP field emission computer-controlled scanning electron microscope with energy-dispersive X-ray microanalysis (CC-SEM–EDX) was used to determine particle size distribution (PSD) of both samples once mounted by dispersion onto silver foil. A backscattered electron detector was used at 1000 × magnification to acquire an image of the stub surface; the heavy elemental background from the silver foil enables a high contrast black and white image to be obtained. The smallest measurable diameter was 0.5 µm as particles smaller than this are indistinguishable from imperfections in the silver foil surface. EDAX Genesis software is used to control the instrument, generating a series of images spiralling out from the centre of the stub, essentially mapping it. The program recognises particles based on their dark colour relative to the background, and greyscale sensitivity is programmed by the operator beforehand. The program measures the *x* and *y* ferets of a particle based on a number of pixels.

Particle size can be represented by average diameter, calculated from the two-dimensional projection of a given particle. Equation 2, as used by Potgieter-Vermaak et al. (2012), shows how average particle diameter is calculated for each particle where *D*_p_ is average particle diameter, *D*_max_ is maximum feret diameter, *D*_min_ is minimum feret diameter.

Average particle diameter

2 $$D_{\text{p}} = \sqrt[3]{{(D_{\hbox{max} } \times D_{\hbox{min} }^{2} )}}$$

### CC-SEM–EDX clustering

Clustering was carried out using the same instrumentation technique as described in “Particle size analysis” section. Each particle identified is exposed to an acquisition of 15 s at 25 kV giving metal concentrations as percentage of each particle based on signal peaks at discrete KeV values indicative of either *Kα* emission or *Lα* emission. Elemental data were then separated into clusters based on mole fractions per element, as described by Kandler et al. (2011). Clusters for these data are defined in Table 1, along with mole fraction criteria. AE refers to total mole fraction; in each case, the contributions of C, O and Ag have been subtracted.

**Table 1:** SPA clusters and criteria

Cluster	Criteria
Silicates	Si/AE > 0.2, Mg/Si < 1.33, Al/Si < 1.33, Fe/Si < 0.5, Ti/Si < 0.5, Na/Si < 0.7
Calcites	Ca/AE > 0.5, Mg/Ca < 0.33, Si/Ca < 0.5, S/Ca < 0.25, P/Ca < 0.15
Ti-rich	Ti/AE > 0.3, Mg/Ti < 1, Al/Ti < 1, Fe/Ti < 1, Si/Ti < 1, S/Ti < 1, Na/Ti < 1
Fe-rich	Fe/AE > 0.15, Si/Fe < 1.05, Ti/Fe < 1.3
Al-rich—not aluminosilicates	Al/AE > 0.15, Si/AE < 0.2, Ti/Al < 1.3
Trace containing	(Cu + Cr + Mn + Ni + Pb + Sn + V+Zn)/AE > 0.05

For the CC-SEM–EDX clustering, another aliquot of the < 38 µm fraction was used which was not exposed to water in the manner described in “Particle size analysis”. This was done in order to minimise any loss of water-soluble metals. Particles have been clustered in accordance with Table 1, with extra criteria added to group particles with a diameter of < 10 µm.

### Acid-soluble metals determination

250 mg ± 10 mg of each < 10 µm separated sample was analysed for its acid-soluble metal content by dissolution in *aqua regia* (7.5 cm^3^ HCl, 2. cm^3^ HNO_3_); three extractions were carried out per sample. This process was facilitated with a 1600 W CEM Mars 5 Microwave equipped with Teflon digestion tubes. Analysis was carried out using a Varian Vista MPX ICP-OES with SeaSpray nebuliser. Initial system stability checks were carried out with a 5 mg cm^−3^ solution of Mn during the torch alignment whereby the upper values must be in excess of 300,000 counts per second, and sample analysis exclusively used the axial position. A four-point linear calibration was achieved for each element; a calibration coefficient of > 0.99 for all elements was determined. Method verification was carried out using a certified reference material (CRM), river clay sediment LGC6139. 500 mg ± 10 mg of CRM was weighed out in triplicate, digested using aqua regia and analysed using ICP-OES. Determined concentrations of available metals were within 4% of the certified mean concentrations. Five replicates were carried out per sample.

### Metal dissolution determination

For the extraction method, 150 mg ± 10 mg of the < 10 µm particles was measured into a PET centrifuge tube along with 15 cm^3^ of artificial lysosomal fluid (ALF). ALF (Colombo et al. 2008) was selected as an appropriate representation of the lung environment cells encounter following contact with the macrophage immune response (Wiseman and Zereni 2014). The mixture was then placed in an incubator shaker for 24 h. This was deemed an appropriate length of time to expose the samples to ALF, as residence time for particles in the lung is still somewhat disputed by the scientific community. Despite this, the aim of this paper is to investigate the use of larger particles as a proxy for respirable particles so extraction time should not be important. The analysis method for the ALF-extracted samples was the same as for the acid-extracted samples. Al, As, Cr, Cu, Fe, Mn, Ni, Pb, Sn and Zn were quantified in each sample. These particular metals were selected to give an insight into the effective change for metals deemed to be of both crustal and anthropogenic origins (Alomary and Belhadj 2007; Gunawardana et al. 2012).

## Results

### Particle size analysis

**Fig. 2:**
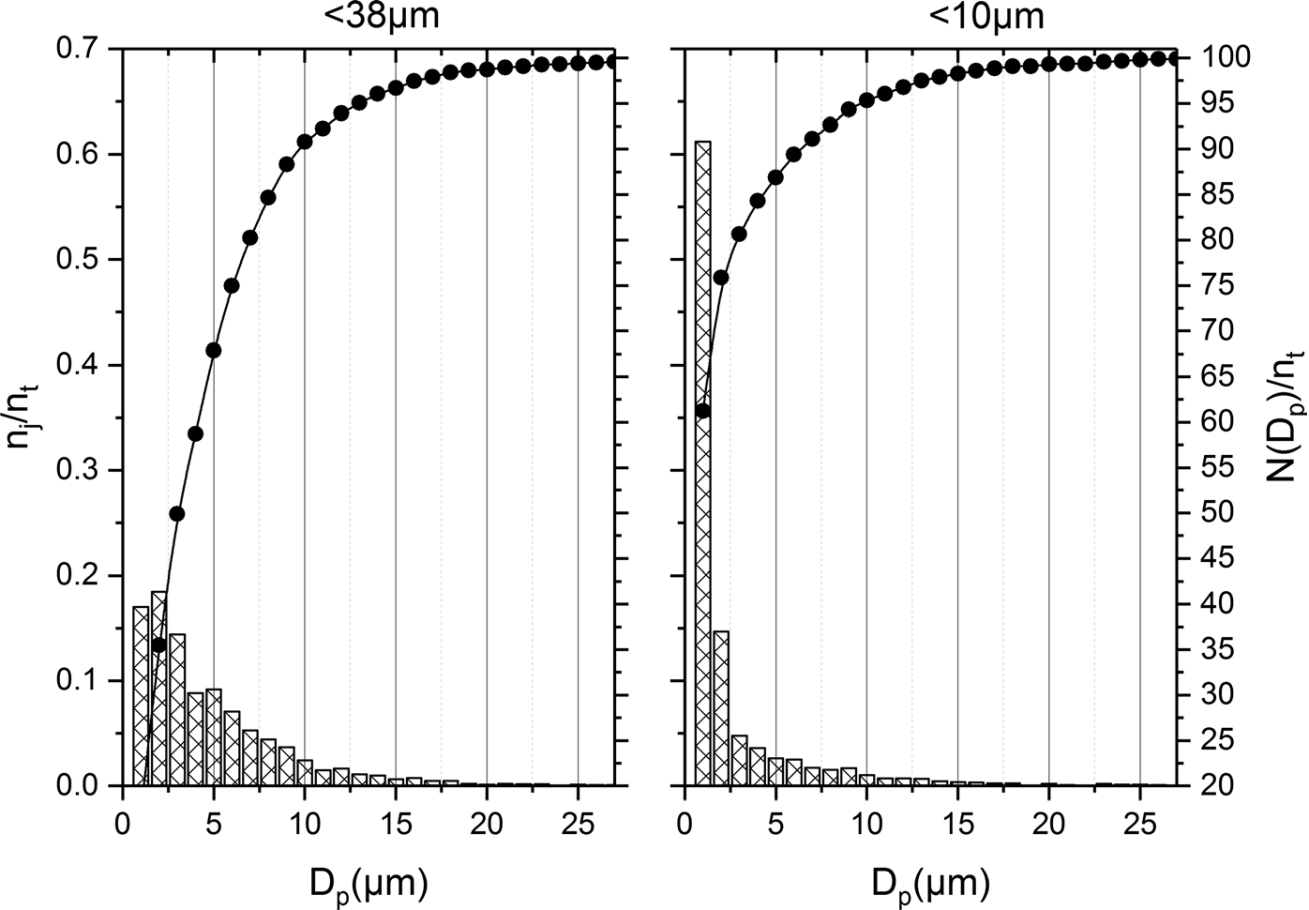
Particle size distributions from each fraction, where *n*_*j*_/*n*_*t*_ indicates the fraction of particles present in that size range and *N*(*D*_p_)/*n*_*t*_ indicates the cumulative distribution

Particle size was determined using CC-SEM–EDX. A total of 1500 particles were analysed for each of the two fractions. Figure 2 shows particle size distribution as determined by CC-SEM–EDX for the < 10 µm and < 38 µm fractions. The figure divides particles into bins based on particle size, starting with 0.5–1.5 µm and then 1.5–2.5 µm, etc., presented along the *x*-axis as average particle diameter (*D*_p_). The primary *y*-axis shows the number of particles in each bin divided by the total number of particles (*n*_*j*_/*n*_*t*_). Presenting particle size distribution data in this manner is common for particulate matter (Chen et al. 1997; Lin et al. 2005; Potgieter-Vermaak et al. 2012; Yue et al. 2013) and RD (McKenzie et al. 2008); these studies tend to log the number of particles in each bin. The authors felt that logging the number of particles in each bin failed to give a clearer visual representation of the particle size distribution. The secondary *y*-axis of Fig. 2 shows a cumulative distribution of particles, by summing the number of particles in each preceding bin, as a percentage of the total number of particles (*N*(*D*_p_)/*n*_*t*_). The cumulative distribution is included to give an indication of the type of distribution observed. Both the < 38 µm and the < 10 µm fractions showed somewhat of a log-normal distribution; however, there are too many smaller particles to fit this distribution with statistical confidence. It is observed that the number of particles smaller than 1.5 µm dropped off in the < 38 µm fraction, but carried on increasing in the < 10 µm fraction. It is possible that this was caused by the separation technique failing to remove some particles between 1.5 and 10 µm from the < 38 µm, therefore under representing them in the < 10 µm fraction.

Geometric median and geometric standard deviation are calculated using equations given by Potgieter-Vermaak et al. (2012). The geometric median particle size in the < 10 µm fraction is 1.7 µm (geometric standard deviation 2.3 µm); 94.3% of particles were smaller than 10 µm. In the < 38 µm fraction, the geometric median particle size was 3.5 µm (geometric standard deviation 2.3 µm); 90.4% of particles were smaller than 10 µm. These results show that a good separation efficiency was achieved with this technique and that a significant portion of the particles larger than 10 µm have been removed. The results fail to report particles smaller than 0.5 µm as these particles could not be quantified by the analytical techniques used in this study. The distribution of particles observed here in the < 10 µm fraction was similar to that reported by McKenzie et al. (2008). In both cases, particles were measured down to a size of 0.5 µm, albeit using different instrumentations. Interestingly, results from this study showed a greater exponential skew towards the smallest particles. This could possibly be due to differences in sites or the vacuum collection method used by Mckenzie et al. (2008). The large number of small particles observed here in the < 38 µm fraction supports the concept that RD inherently contains a large number of small particles (Kong et al. 2012). This is a crucial observation with respect to the aim of this study; a significant portion of road dust particles, by number of particles, are capable of entering the thoracic cavity.

### CC-SEM–EDX clustering

Table 2 shows cluster data for the original < 38 µm fraction, and for particles within this sample that have a diameter of < 10 µm, the portion within each cluster observed is presented as a percentage, in order to normalise the number of particles of a given observed size. A large number of particles were found to match the criteria for more than one cluster. These mixed clusters are also defined in Table 2.

**Table 2:** Abundance of clusters present in the 1500 particles of < 38 µm and < 10 µm fraction

Cluster	Clusters present in particles < 38 µm (%)	Clusters present in particles < 10 µm (%)
Silicates	57.7	56.3
Silicates and calcites mixed	0.3	0.4
Silicates and trace mixed	6.9	7.3
Calcites	0.4	0.4
Calcites and Al-rich mixed	4.2	4.3
Calcites, Al-rich and trace mixed	7.1	7.3
Ti-rich	0.2	0.1
Fe-rich	2.5	2.3
Fe-rich and trace mixed	0.3	0.3
Al-rich	1.6	1.3
Al-rich and trace mixed	8.2	7.4
Trace	7.2	7.3
Undefined	3.4	5.2

Table 2 shows markedly similar clustering data between the two size fractions. The largest absolute difference between the two fractions was the silicates cluster, where the < 38 µm fraction contained 1.4% more silicates than the < 10 µm fraction. Calcites, Ti-rich, Fe-rich, Al-rich and trace clusters were all within 0.3% of each other with respect to the different size fractions. There was a slightly more noticeable difference between the mixed clusters, where the differences between the size fractions ranged up to 0.8%. This could suggest that in both samples particles are commonly present as aggregated collections of particles, in agreement with previous research on the topic (Thorpe et al. 2007).

The undefined row of Table 2 indicates that particles fit none of the clustering criteria. In most circumstances, this classification was attributed to highly carbonaceous particles, containing trace amounts of various other elements. It is observed that there is a significantly larger portion of undefined particles in the < 10 µm size fraction, which could be the result of a larger fraction of soot/vehicle exhaust particles.

### Bulk elemental concentration

In order to further verify if the < 38 µm can act as a predictor for bioaccessible behaviour of smaller particles, the bulk elemental concentration of the particles was investigated. Results from the acid digestion experiment indicated a significant difference in metal concentrations observed between the < 38 and < 10 µm fractions. Elemental concentrations were increased in the < 10 µm fraction for each element quantified. Table 3 represents the data from both fractions and includes the % increase from < 38 to < 10 µm.

**Table 3:** Acid-soluble metal concentrations

	Al	As	Cr	Cu	Fe	Mn	Ni	Pb	Sn	Zn
<10 µm (mg/kg)	212,700 ± 7.9%	49.7 ± 8.6%	674.8 ± 5.1%	7224 ± 0.9%	397,000 ± 1.1%	6180 ± 0.8%	258.1 ± 6.7%	3290 ± 0.8%	535.5 ± 1.4%	11,240 ± 0.9%
<38 µm (mg/kg)	140,100 ± 3.6%	30.0 ± 11%	636.8 ± 3%	4576 ± 1.6%	355,400 ± 1.5%	5788 ± 1.6%	209.7 ± 3.8%	2320 ± 0.4%	461.3 ± 1.6%	7564 ± 1.6%
Increase (%)	51.8	66.3	6.0	57.8	11.7	6.8	23.1	41.8	16.1	48.6

Elemental concentrations in the < 10 µm fraction showed the trend Fe > Al > Zn > Cu > Mn > Pb > Cr > Sn > Ni > As. The same pattern was observed in the < 38 µm fraction, with the exception of Mn, which was more abundant than copper. Albuquerque et al. (2017) concluded a similar pattern of metal concentration of PM_10_, Zn > Cu > Pb > Mn > Cr > Ni > As, the notable difference being the increased Pb concentration relative to Mn. Other studies investigating metal concentrations of PM_10_ also indicated a similar order, again with Pb being more abundant than Mn (Lim et al. 2010). It is worth noting that specific metal concentrations in terms of absolute concentration and relative to other metals are very dependent on site. It is also worth acknowledging that the literature quoting concentrations of metals within airborne PM_10_ will generally be reported in units of mass per volume (of air), as collection on filters is the most common method of quantification.

Such large differences in metal concentrations between the fractions may appear to conclusively disprove any possibility that the two samples may be comparable. However, it is documented that the largest constituent of RD is Si-containing particles, and therefore, it is expected that the concentration of Si increases in larger grain fractions (Brown et al. 2015). It is feasible that the difference between the two samples may be the significantly larger concentration of Si in the < 38 µm fraction. If that was the only reason, one would expect a fairly constant difference across the elements, which is not observed. On the other hand, as we report on acid-soluble elemental profiles and not complete digestion of the sample, we can also attribute some of these differences to selective or incomplete digestion of some elements, thereby explaining the variation in differences observed. The larger < 38 µm fraction will evidently show lower solubility of the silicate-containing particles than the < 10 µm fraction which has a larger surface area-to-volume ratio. It is also well known that the larger fraction of dust is more silicate-rich than the smaller fractions. Furthermore, the differential dissolution between the two fractions could also mean that some of the metals are bonded in a different matter, for example absorbed on to the surface of other particles, resulting in higher yields for the smaller fraction.

An alternative approach to assessing the difference between the aqua regia-soluble metals in the samples, we can refer back to the SEM–EDX work originally carried out as a means of ascertaining particle size distribution. Recalculating the percentage weight (%Wt) composition for each particle once C, O and Si have been removed, we can compare quantities of each remaining element present in each sample. A full suite of crustal and trace elements quantified by SEM–EDX were left in the %Wt equation to increase accuracy. Distribution of each element also quantified by ICP-OES was found to be skewed left (Al, As, Cr, Cu, Fe, Mn, Ni, Pb, Sn and Zn). Therefore, the Mann–Whitney *U* test was used to compare each of them between the < 10 and < 38 µm faction, the null hypothesis (H_0_) being that the samples are statistically not the same. With the exception of Fe, Zn and As, it was found that we can accept the alternative hypothesis for all other elements. In the case of As, the SEM–EDX data indicated too few As-containing particles in either fraction to perform a Mann–Whitney *U* test. With regard to Fe and Zn, the Mann–Whitney *U* test indicated that these elements were more enriched in the < 10 µm fraction.

### ALF-soluble metals’ results

Since the elemental concentrations of the two fractions, as determined by SEM, showed significantly similar profiles, the last step was to test the bioaccessible profiles of the two fractions. Concentrations of ALF leachate were normalised to mg/kg, in the same way as the acid digested samples were. For simplicity, the data can then be represented as bioaccessibility (%) for each element using Eq. 3. Results for each metal are presented in Table 4.

**Table 4:** Metal leachate profiles from both fractions displayed as percentage bioaccessibility

	Al	As	Cr	Cu	Fe	Mn	Ni	Pb	Sn	Zn
< 10 µm	1.74 ± 0.08	10.79 ± 0.1	5.14 ± 0.08	8.39 ± 0.03	3.11 ± 0.08	7.61 ± 0.06	9.93 ± 0.07	7.56 ± 0.05	5.03 ± 0.1	10.08 ± 0.05
< 38 µm	1.56 ± 0.04	12.35 ± 0.12	3.33 ± 0.04	5.51 ± 0.03	2.24 ± 0.02	5.64 ± 0.02	8.98 ± 0.04	6.26 ± 0.03	4.57 ± 0.02	8.49 ± 0.02
% Underestimation	10.3	− 14.5	35.2	34.3	28.0	25.9	9.6	17.2	9.1	15.8

Bioaccessibility (%)

3 $${\text{Bioaccessibility}}\ (\% ) = \frac{{\left[ {\text{ALF leachates}} \right]({\text{mg}} {\text{kg}}^{ - 1} )}}{{\left[ {\text{Acid digestion}} \right]({\text{mg}} {\text{kg}}^{ - 1} )}}$$

Standard deviations for each metal are given as calculated by propagation of errors (Miller and Miller 2005). The results do seem to indicate a strong similarity between the leaching behaviours of the two samples across the full range of metals. There is an average difference of just 1.35% between any given elements with respect to fraction size. The largest absolute difference is 2.88% for Cu; the smallest is 0.18% for Al. This appears to support the elemental composition results obtained using SEM–EDX, as well as the clustering data, which suggested similar associations of the elements. It is, therefore, not unreasonable to say that the results suggest that the chemical composition of the two samples is similar in their leaching behaviour in the ALF. Vastly different leaching behaviours between the two samples may suggest that the elements reported on here are differently associated with each other or other elements between the two samples. In general, it is observed that the < 38 µm fraction will underestimate the % bioaccessibility on average by 20%, with notable underestimation of 26–35% for Mn, Fe, Cu and Cr. In comparison with other studies, the bioaccessibility in ALF results achieved here for both size fractions is significantly lower than that collected by Wiseman and Zereni (2014), who achieved bioaccessibility around an order of magnitude higher across the suite of metals analysed, generally between 60 and 80% bioaccessible. It should be noted, however, that the particles analysed in this study were PM_10_, PM_2.5_ and PM_1_, which would be expected to leach metals more readily. Pelfrene et al. (2017) carried out ALF dissolution tests on a range of certified reference materials. Results indicated large variability in dissolution of metals between each of the reference materials; % bioaccessibility of Mn was found to be 5.5% (± 0.1), 44.3% (± 0.2) and 46.8% (± 2.6) amongst the three analysed reference materials; this would indicate that the chemical composition of a specific sample affects the ability of metals to leach into simulated biological fluids. The similarity in bioaccessibilities of the two samples analysed in this study indicates that the metals may be present in the same chemical composition and thus are representative of one another.

## Conclusions

The aim of this study was to assess the possibility of using larger size fractions of urban particles to act as a proxy for respirable particles, in terms of metal concentrations and their in vitro profile. The data show that there are a large number of similarities between the leaching behaviours of the two fractions and that similarities also exist in the clustering profile. Despite this, the absolute metal concentrations between the fractions are quite dissimilar with regard to their aqua regia-soluble behaviour. One explanation could be that silica-rich particles are more concentrated in large particles and thus dilute metal concentrations therein. Another explanation could be that metals within the smaller particles are more efficiently solubilised. Similarities in leaching behaviour in artificial lysosomal fluid and elemental association as determined by clustering CC-SEM–EDX data, as presented in this study, suggest that the larger fraction is a precursor to the smaller. We would like to propose that the larger fraction has a significant presence of aggregates which ultimately disintegrate to form the smaller fraction, providing particles of similar chemical composition. This conclusion is touched upon in discussion “CC-SEM–EDX clustering” section.

Referring back to the aim, as outlined in introduction section of this paper, it does appear that the < 38 µm fraction could be used as at least a first approximation of metal enrichment in respirable particles for the purpose of in vitro tests. It therefore enables researchers to easily use a larger sample size and thus provide more representative results. Further work on the topic using intra-particle analytical techniques, to elucidate the chemical composition of metals, for example Raman spectroscopy, could broaden the conclusions drawn here.

## References

[CR1] Albuquerque M, Coutinho M, Rodrigues J, Ginja J, Borreo C (2017). Long-term monitoring of trace metals in PM10 and total gaseous mercury in the atmosphere of Porto, Portugal. Atmospheric pollution research.

[CR2] Alomary AA, Belhadj S (2007). Determination of heavy metals (Cd, Cr, Cu, Fe, Ni, Pb, Zn) by ICP-OES and their speciation in Algerian Mediterranean Sea sediments after a five-stage sequential extraction procedure. Environmental Monitoring and Assessment.

[CR3] Behrooz R, Esmaili-Sari A, Bahramifar N, Kaskaoutis DG (2017). Analysis of the TSP, PM_10_ concentrations and water-soluble ionic species in airborne samples over Sistan, Iran during the summer dusty period. Atmospheric Pollution Research.

[CR4] Blake GR, Hartage KH (1986). Particle density. Methods of soil analysis part 1—physical and mineralogical methods.

[CR5] Boisa N, Entwistle J, Dean JR (2014). A new simple; low-cost approach for generation of the PM10 fraction from soil and related materials: Application to human health risk assessment. Analytica Chemica Acta.

[CR6] Brown A, Barrett JES, Robinson H, Potgieter-Vermaak S (2015). Risk assessment to particulate output of a demolition site. Environmental Geochemistry and Health.

[CR7] Chen Shui-Jen, Shi-Hu Liao, Jian Wei-Jain, Lin Chih-Chung (1997). Particle size distribution of ambient aerosol carbons in ambient air. Environment International.

[CR8] Colombo C, Monhemius AJ, Plant JA (2008). Platinum, palladium and rhodium release from vehicle exhaust catalysts and road dust exposed to simulated lung fluids. Ecotoxicology and Environmental Safety.

[CR9] EEA. (2016). http://www.eea.europa.eu/themes/air/air-emissions-data. Accessed 12 June 2019.

[CR10] Gunawardana C, Goonetilleke A, Egodawatta P, Dawes L, Kokot S (2012). Source characterisation of road dust based on chemical and mineral composition. Chemosphere.

[CR11] Harrison RM, Smith DGT, Pio CA, Castro LM (1997). Comparative receptor modelling study of airborne particulate pollutants in Birmingham, United Kingdom, Coimbra (Portugal) and Lahore (Pakistan). Atmospheric Environment.

[CR12] Haynes (2018). CRC handbook of chemistry and physics.

[CR13] Hien PD, Binh NT, Truong Y, Ngo NTN (1999). Temporal variations of source impacts at the receptor, as derived from particulate monitoring data in Ho Chi Minh City, Vietnam. Atmospheric Environment.

[CR14] Kandler K, Leike K, Benker N, Emmel C, Kupper M, Muller-Ebert D, Scheuvens D, Schladitz A, Schutz L (2011). Electron microscopy of particles collected at Praia, Cape Verde, during the Saharan Mineral Dust Experiment: particle chemistry, shape, mixing state and complex refractive index. Tellus.

[CR15] Kong S, Lu B, Ji Y, Zhao X, Bai Z, Xu Y, Liu Y, Jiang H (2012). Risk assessment of heavy metals in road and soils dust within PM2.5, PM10, and PM100 fraction is Dongying city, Shandong province, China. Journal of Environmental Monitoring.

[CR16] Landis MS, Pancras JP, Graney JR, White EM, Edgerton ES, Legge A, Percy KE (2017). Source apportionment of ambient fine and coarse particulate matter at the Fort McKay community site, in the Athabasca Oil Sands Region, Alberta, Canada. Science of the Total Environment.

[CR17] Lim JM, Lee JH, Moon JH, Chung YS, Kim KH (2010). Airborne PM10 and metals from multifarious sources in an industrial complex area. Atmospheric Research.

[CR18] Lin CC, Chen SJ, Huang KL, Hwang WI, Chang-Chien GP, Lin WY (2005). Characteristics of metals in nano/ultrafine/fine/coarse particles collected beside a heavily trafficked road. Environmental Science and Technology.

[CR19] McKenzie ER, Wong CM, Green PG, Kayhanian M, Young TM (2008). Size dependant elemental composition of road-associated particles. Science of the Total Environment.

[CR20] Padoan E, Rome C, Ajmone-Marsan F (2017). Bioaccessibility and size distribution of metals in road dust and road side soils along a peri-urban transect. Science of the Total Environment.

[CR21] Pelfrene A, Cave MR, Wragg J, Douay F (2017). *In vitro* investigations of human bioaccessibility from reference materials using simulated lung fluids. Environmental research and public health.

[CR22] Potgieter-Vermaak S, Hoiremans B, Anaf W, Cardell C, Van Grieken R (2012). Degradation potential of airborne particulate matter at the Alhambra monument: A Raman spectroscopic and electron probe X-ray microanalysis study. Raman Spectroscopy.

[CR23] Rohr AC, Kamal A, Morishota M, Mukherjee B, Keeler GJ, Harkema JR, Wagner JG (2011). Altered heart rate variability in spontaneously hypertensive rats is associated with specific particulate matter components in Detroit, Michigan. Environmental Health Perspective.

[CR24] Shahsavani A, Naddafi K, Haghighifard NJ, Mesdaghinia A, Yunesian M, Nabizadeh R, Arhami M, Yarahmadi M, Sowlat MH, Ghani M, Jafari AJ, Alimohamadi M, Motevalian SA, Soleimani Z (2012). Characterization of ionic composition of TSP and PM10 during the Middle Eastern dust (MED) storms in Ahvaz, Iran. Environmental Monitoring and Assessment.

[CR25] Thorpe AJ, Harrison RM, Boulter PG, McCrae ISM (2007). Estimation of particle resuspension source strength on a major London road. Atmospheric Environment.

[CR26] Tian F, Bonnier F, Casey A, Shanahan A, Byrne H (2014). Surface enhanced Raman scattering with gold nanoparticles: Effects of particular shape. Analytical Methods.

[CR27] Wang J, Zhou M, Liu B, Wu J, Peng X, Zhang Y, Han S, Feng Y, Zhu T (2016). Characterization and source appointment of size segregated atmospheric particulate matter collected at ground level and from the urban canopy in Tianjin. Environmental Pollution.

[CR28] Whittaker A, Berube K, Jones T, Maynard R, Richards R (2004). Killer smog of London, 50 years on: Particle properties and oxidative capacity. Science of the Total Environment.

[CR29] WHO. (2009). Global health risks, mortality and burden of disease attributable to selected major risks. http://www.who.int/healthinfo/global_burden_disease/GlobalHealthRisks_report_full.pdf. Accessed 12 June 2019.

[CR30] Wiseman C, Zereni F (2014). Characterizing metal(loid) solubility in airborne PM10, PM2.5 and PM1 in Frankfurt, Germany using simulated lung fluids. Atmospheric Environment.

[CR31] Worobiec A, Potgieter-Vermaak S, Brooker A, Darchuck L, Van Grieken R (2010). Interfaced SEM/EDX and micro-Raman spectroscopy for characterisation of heterogeneous environmental particles—Fundamental and practical challenges. Microchemical Journal.

[CR32] Wragg J, Cave M, Basta N, Brandon E, Casteel S, Denys S, Gron C, Oomen A, Reimer K, Tack K, Van de Wiele T (2011). An inter-laboratory trial of the unified BARGE bioaccessibility method for arsenic, cadmium and lead in soil. Science of the Total Environment.

[CR34] Yue DL, Hu M, Wang ZB, Wen MT, Guo S, Zhong LJ, Weidensohler A, Zhang YH (2013). Comparison of particle number size distribution and new particle formation between the urban and rural sites in the PRD region, China. Atmospheric Environment.

